# Brain Opioid Activity and Oxidative Injury: Different Molecular Scenarios Connecting Celiac Disease and Autistic Spectrum Disorder

**DOI:** 10.3390/brainsci10070437

**Published:** 2020-07-09

**Authors:** Diana Di Liberto, Antonella D’Anneo, Daniela Carlisi, Sonia Emanuele, Anna De Blasio, Giuseppe Calvaruso, Michela Giuliano, Marianna Lauricella

**Affiliations:** 1 Department of Biomedicine, Neurosciences and Advanced Diagnostics (BIND), University of Palermo, 90127 Palermo, Italy; diana.diliberto@unipa.it; 2 Department of Biological, Chemical and Pharmaceutical Sciences and Technologies (STEBICEF), Laboratory of Biochemistry, University of Palermo, 90127 Palermo, Italy; anna.deblasio@unipa.it (A.D.B.); giuseppe.calvaruso@unipa.it (G.C.); michela.giuliano@unipa.it (M.G.); 3 Department of Biomedicine, Neurosciences and Advanced Diagnostics (BIND), Institute of Biochemistry, University of Palermo, 90127 Palermo, Italy; daniela.carlisi@unipa.it (D.C.); sonia.emanuele@unipa.it (S.E.)

**Keywords:** celiac disease, Autistic Spectrum Disorder, opioids, oxidative stress, mitochondrial damage

## Abstract

Celiac Disease (CD) is an immune-mediated disease triggered by the ingestion of wheat gliadin and related prolamins from other cereals, such as barley and rye. Immunity against these cereal-derived proteins is mediated by pro-inflammatory cytokines produced by both innate and adaptive system response in individuals unable to adequately digest them. Peptides generated in this condition are absorbed across the gut barrier, which in these patients is characterized by the deregulation of its permeability. Here, we discuss a possible correlation between CD and Autistic Spectrum Disorder (ASD) pathogenesis. ASD can be induced by an excessive and inappropriate brain opioid activity during the neonatal period. Cereal-derived peptides produced in celiac patients cross the blood–brain barrier and bind to endogenous opioid receptors interfering with neurotransmission and generating deleterious effects on brain maturation, learning and social relations. Moreover, an increase in oxidative stress and a decrease in the antioxidant capacity, as well as an extended mitochondrial impairment in the brain, could represent a possible connection between ASD and CD. Therefore, we critically discuss the proposed relationship between ASD and CD and the possible usefulness of a gluten-free diet in ASD patients.

## 1. Introduction

Celiac disease (CD) is an immune-mediated reaction to gluten, a protein complex made of gliadin and glutenin proteins [1], affecting genetically predisposed individuals. The prevalence of CD is estimated to be 0.5–1% of the general population and the incidence of disease has increased in the last few decades [2,3]. Gastrointestinal symptoms are typical features of CD, together with malabsorption and consequent weight loss, fatigue and nutrient deficiencies, diarrhea and bloating. Celiac patients have anti endomysial and anti tissue transglutaminase antibodies in their blood, with a high specificity and sensitivity [4]. Currently, the gold standard for CD diagnosis is endoscopy with duodenal biopsy [5]. Histological changes, including intraepithelial lymphocytosis, crypt hyperplasia, and varying degrees of villous atrophy, are graded according to a classification system, proposed by Marsh and modified by Oberhuber (Marsh I-IIIc) [6].

Beyond gastrointestinal symptoms, CD patients can display other symptoms related to the central nervous system dysfunction, including epilepsy, ataxia and migraine [7], as well as neurological symptoms [8]. A recent clinical case shows how schizophrenia could be a symptom of CD, resolving with a gluten-free diet treatment [9]. Moreover, comorbidity between CD and Autistic Spectrum Disorder (ASD) has been reported [10], although the mechanism of such a correlation needs to be better clarified. ASD are highly heterogeneous neurodevelopmental disorders, characterized by different symptomatology and causes [11,12]. This heterogeneity may explain the difficulty to establish efficient therapeutic approaches. The identification of novel etiological traits may provide therapeutic indications for a specific category of patients. In this perspective, understanding the mechanisms underlying the comorbidity between CD and ASD is important to recommend a more specific therapeutic approach to ASD patients with CD.

Many different hypotheses, some very popular and others more controversial, have been proposed as possible underlying events in ASD. Some of these focused on an altered excitatory-inhibitory balance of neural systems [13], recently regarded as a crucial framework to unveil aspects related to pathophysiology as well as to identify targeted interventions for the treatment of ASD patients [14].

Another hypothesis considered the role of an unbalanced gut microbiota (dysbiosis) as a comorbidity in young ASD patients manifesting the prevalence of gastrointestinal problems in a range of 23–85% patients [15,16]. This observation was also sustained by recent data, providing that the transplantation of gut microbiota from ASD patients in mice promotes the development of characteristic autistic behaviors [17], albeit differences between human and murine microbiota exist and such an aspect also has to be considered [18]. A large amount of evidence has also pushed to consider the existence of a microbiota–gut–brain axis, although a causative relationship between ASD and gut microbiota remains not completely well established so far [19].

Another relevant hypothesis was formulated by the exploratory work of Panksepp and colleagues, named the Brain Opioid Theory of Social Attachment. On this subject, Pellisier in his μ opioid receptor balance model sustained that an unbalanced μ opioid receptor (μOR) function can be sufficient to hamper social behavior, contributing to ASD [20]. Indeed, the ablation of μOR can also favor the loss of social behaviors [21,22]. On the contrary, in the State-dependent μ-Opioid Modulation of SOcial Motivation (SOMSOM) model, the initial social context is regarded as a key determinant of the effects of opioids on social behavior [23].

More specifically, considering that the scenario of hypotheses for ASD is quite complex and, under certain aspects, controversial, the current review aimed at providing an overview of some hypotheses connecting CD and ASD. Here, in particular, we analyze the role of brain opioid activity and oxidative injury as causative events connecting CD and ASD under an exquisitely biochemical point of view. We believe that a deeper investigation of these aspects could provide new predictive biochemical markers and better clarify the controversial role of a gluten-free diet for those ASD patients bearing CD symptoms.

## 2. Celiac Disease and Non-Celiac Gluten Sensitivity

CD is a human autoimmune-like disorder characterized by the chronic inflammation of the small intestine with environmental, genetic and immunologic factors, playing a role in its pathogenesis [24]. The immune system of CD patients reacts to some proteins, called prolamins, contained in wheat (gliadin), rye (secalin) and barley (horedin). In addition, a few of them also have an immune reaction to oats, which contain avenin [25]. 

A typical feature observed in CD patients is malabsorption. This is a critical condition in CD pathogenesis resulting from the T-cell mediated damage of the intestinal mucosa and it is histologically represented by villous atrophy, crypt hyperplasia, and the infiltration of lymphoid cells both in the epithelium and in the lamina propria [24].

The inflammatory process underlying CD pathogenesis involves the activation of the innate as well as the adaptive immune system [26], being predominantly characterized by a strong T helper (Th)1 response with the production of pro-inflammatory cytokines, such as interferon gamma (IFN-γ) and tumor necrosis factor alpha (TNF-α) [27]. The innate immune response is responsible for triggering the inflammatory cascade [28]. Confirming this hypothesis, innate pro-inflammatory cytokines, including interleukin-15 (IL-15), are upregulated after the destruction of epithelial cells by toxic gliadin peptides, such as 19-mer [29,30]. Moreover, IL-15 and interferon alpha (IFN-α) were demonstrated to be produced following gliadin ingestion in untreated CD patients. Furthermore, interleukin-8 (IL-8) seems to be released by the epithelium and by the immune cells of CD patients, being a strong chemo-attractive agent for neutrophils [31,32]. The literature data [1] also demonstrate that a potent inflammatory response is triggered by macrophages, which are activated following gliadin exposure. The intestinal epithelium of CD patients seems to mediate the downregulation of macrophage response, being instead more responsive to epithelium-derived signals when compared to non-CD subjects. Taken together, these data suggest the existence of a crosstalk between the intestinal epithelium and immune cells, whose deregulation is probably involved in the loss of gluten tolerance, as well as in CD development in genetically predisposed individuals. Beyond gluten ingestion, an individual, indeed, possesses a genetic predisposition to develop CD. Such a condition has been associated to DQ2 and DQ8 Human Leukocytes Antigens (HLA), two alleles commonly found in CD patients. Contrarily, the absence of HLA antigens reduces the risk of developing the disease, even after a gluten rich diet exposure. 

Chronic inflammation is driven by the adaptive immune response that is induced by toxic gliadin peptides, such as 33-mer, which, entering the lamina propria, are presented by the HLA class II molecules DQ2 and DQ8 to Th1 cells. Th17 cells are also involved in CD pathogenesis and development, as the interleukin-17A (IL-17A) gene was found more highly expressed in duodenal biopsies of CD patients than in the controls [33,34]. Chronic inflammation in CD patients has also been correlated with peroxisome proliferator-activated receptor gamma (PPARγ) down-regulation. PPARγ is a transcription factor which exerts protective effects in immune-mediated diseases, due to its ability to negatively regulate the expression of gene coding for pro-inflammatory cytokines [35]. Notably, the low expression of PPARγ and high expression of transglutaminase were observed in the duodenal mucosa of children with CD and this event has been correlated with some toxic gliadin peptides. In particular, p31–43 peptides induce oxidative stress which promote the tranglutaminase-dependent proteasomal degradation of PPARγ [36].

Data available so far have also proved that many factors could contribute to CD onset in genetically high-risk individuals, including method of infant feeding [37], of birth (vaginal vs. caesarean section) [38], a chronic inflammatory immune response to gliadin [3] and its deficient suppression by Treg cells [39], time of gluten introduction into the diet [22], infections occurrence and also the presence of several non-HLA genes [40]. In addition, the risk of developing CD is higher in the presence of other autoimmune diseases or other pathologies, including Down syndrome, Williams syndrome, Turner syndrome and also cystic fibrosis [28,41,42,43]. The treatment of CD is primarily a gluten-free diet (GFD), which requires significant patient education, motivation and follow-up [44]. 

However, the picture on this landscape could get more complicated. CD, together with Wheat Allergy (WA) and Non-Celiac Gluten Sensitivity (NCGS), represent different Gluten-Related Disorders (GRD) [45,46]. GRD is a broad-spectrum term used for describing all the adverse reactions generated by the ingestion of gluten-containing food. CD is an immune-mediated enteropathy characterized by the presence of specific autoantibodies against tissue transglutaminase 2 (anti-TG2) and endomysium (EMA). WA is a classic food allergy triggered by wheat, and not only gluten, ingestion that leads to type I and type IV hypersensitivity. IgE immunoglobulins play a crucial role in WA disorder [47], however non-IgE-mediated WA also exists, being difficult to distinguish from NCGS [48]. 

Finally, NCGS was defined by the 2015 Salerno Experts’ Criteria [49,50] as a clinical state characterized by both intestinal and extraintestinal symptoms arising shortly after the ingestion of gluten-containing foods, with a recovery on a gluten-free diet (GFD) in patients which do not manifest neither CD nor WA. The most common symptoms of NCGS are Irritable Bowel Syndrome (IBS)-like, such as bloating, diarrhea, abdominal pain and dyspepsia. Nevertheless, extra intestinal manifestations are often reported, including fatigue, headache, dermatitis (eczema or skin rush), cognitive impairment, depression and anemia [51,52]. Moreover, NCGS patients are characterized by the absence of celiac-specific antibodies and villous atrophy, by a variable HLA status and anti-gliadin antibodies (AGA). As the triggers of an adverse immune response, cereal proteins could include fractions other than gluten, such as amylase–trypsin inhibitors (ATIs) and Fermentable, Oligosaccharides, Disaccharides and Monosaccharides and Polyols (FODMAPs). Some experts proposed to define this entity as “non-celiac wheat sensitivity”, although this terminology excludes the possibility that other gluten-containing cereals (rye, barley) may be a trigger for NCGS symptomatology [11,49].

NCGS prevalence in the general population is still variable, mainly because many patients are self-diagnosed, reporting symptomatic benefit with a gluten-free diet. The double-bind placebo-controlled crossover gluten challenge, proposed by Salerno Experts’ Criteria, remains the “gold standard” for NCGS diagnosis [49]. However, the reported prevalence of Self-Reported Non-Coeliac Wheat Sensitivity (SR-NCWS) ranges between 4.3% and 14.9%, with a pooled global prevalence of about 10% [53]. 

Interestingly, a gut mucosal barrier dysfunction seems to be involved in the pathogenesis of CD as well as NCGS [54]. Although the pathogenic mechanisms leading to the onset of NGCS are not clearly understood, the current opinion is that gluten exposure increases gut permeability both in NCGS and CD patients, albeit the underlying molecular mechanisms are not so similar. In this area of research, many studies tried to explore new possible molecular targets, focusing on Claudins (CLDNs). It is well known that Claudins are integral tight junction (TJ) components, which are essential for cell–cell adhesion maintenance in epithelial monolayers and can regulate intestinal epithelium permeability [43,45]. For example, CLDN1 and CLDN4 seem to be correlated to a TJ-dependent decreased permeability [46]. Confirming these data, NCGS mucosa expresses high levels of CLDN4 transcripts when compared to CD or controls, while the expression of other CLDN genes or other genes associated with TJ functions, such as zonula occludens 1 and occludin, is similar in NCGS or CD mucosa when compared to controls [55]. This suggests the existence of distinct clinical traits qualifying both NCGS and CD patients correlated to both different mucosal barrier properties and CLDN4 gene expression profiles [46].

In particular, it was demonstrated that gliadin peptides can interact with gut epithelial cells through the CXCR3 receptor, leading to the release of zonulin, a molecule involved in gut permeability, and resulting in antigen trafficking from intestinal lumen to the lamina propria. Such an observation appears to be particularly relevant, since a higher expression of CXCR3 receptor was found in active CD than in non-CD intestinal tissues [47]. This was shown not only in CD patients but also in non-CD patients that reported diarrhea-predominant Irritable Bowel Syndrome (IBS) with gastrointestinal symptoms after gluten ingestion [49].

Barbaro et al. [56] reported that serum zonulin can be considered a diagnostic biomarker in NCGS and, if associated with demographic and clinical data, could differentiate NCGS from IBS-D patients with 81% accuracy. However, the levels of zonulin were reduced only in NCGS HLA-DQ2/8-positive patients after six months of GFD, suggesting that zonulin can not represent a diagnostic biomarker for all NCGS patients. Supporting the presence of extraintestinal symptoms, it is well known that not only CD, but also patients positive for circulating AGA in the absence of CD, can present neurological disorders, such as ataxia, peripheral neuropathy, epilepsy, encephalopathy and myopathy, although a study performed on 562 patients AGA positive with neurological disorders demonstrated the absence of enteropathy in most of them [57]. Nevertheless, other experimental evidence has highlighted that a significant percentage of neurological dysfunctions with unknown etiology could be related to gluten ingestion [58]. Specifically, the so called “gluten ataxia” in patients with circulating AGA and without any other ataxia pathogenic factor is the most frequent neurological manifestation of both CD and NCGS [57]. Neuropathy associated with AGA positivity is the second most frequent neurological condition related to both CD and NCGS [57], although the significance of AGA in the absence of biopsy-proven intestinal damage is still a matter of debate [59,60]. The cause of neurological symptoms in patients with NCGS remains unknown, even if a recent experimental evidence focuses on immune-mediated mechanisms. Infiltrating lymphocytes were found in the cerebellum of subjects with ataxia and AGA positive, as well as in the peripheral nerve of patients with neuropathy and AGA positive [59,61]. Several findings highlighted the capacity of serum AGA to bind neural tissue, thus cross-reacting with autoantigens with a mechanism of molecular mimicry [59,62]. Alaedini et al. [63], reported the identification of a cross-reactive protein, synapsin I, a neuronal phosphoprotein involved in the regulation of neurotransmitter release. Anti-synapsin reactivity was present in both NCGS and CD patients, but not in control subjects, without AGA. This could explain how, in some NCGS patients, the anti-gliadin antibodies affecting the synapsin I activity, interfere with neurotransmitter release, resulting in neurological dysfunctions. Moreover, this cross-reactivity could also lead to T cell-mediated tissue damage. Among the CD patients, anti-synapsin could be present or not, suggesting that, in autoimmune disorders, Ab reactivity is only a part of the whole pathogenic mechanism causing neurological impacts in CD patients, and the potential pathogenic contribution of anti-synapsin antibodies to neuropathy depends on other concurrent factors, such as the integrity of the blood–nerve or blood–brain barrier and the presence of pro-inflammatory factors. A paper published in 2018 reported a small study performed on 60 patients with neuropathy, mostly without CD, improving after a GFD, with an 89% reduction in the risk of peripheral neuropathic pain [64]. Additionally, patients affected by gluten neuropathy on one year of GFD reported a significant improvement in their neurological symptoms. Nevertheless, this study should be considered with caution because patients were not randomly and blindly recruited, remaining significant even after 29% of celiac patients were not considered for the analysis [65]. Table 1 summarizes the characteristics of CD, the main risk factors and the correlated diseases. 

## 3. Autistic Spectrum Disorder

Autistic Spectrum Disorder (ASD) is a complex neurodevelopmental condition showing clinical signs in children before three years of age [50]. It is a behaviorally well-defined disorder characterized by a qualitative impairment in social and communication skills accompanied by repetitive behaviors or restricted interests [68]. Population-based studies in America and the United Kingdom have reported that the prevalence of ASD is dramatically increasing, becoming a public health problem [66,67] with an incidence rate which increased 4-fold during the last three decades (1:300 in 1996 [67], 1:150 in 2002 [66], 1:68 in 2020 [67]). In 1977, Folstein and Rutter [69] had already hypothesized that the presence of inherited genetic alterations could favor the development of ASD. About 800 genes have been identified that may be implicated in ASD predisposition [70] and, among them, about fifty genes have been associated with a high risk of incidence of the disease. Genes exerting a key role in the onset of ASD include those coding for: adhesion proteins (such as neuroligins, cadherins and neurexins) [71]; synapsin (a phosphoprotein involved in neurotransmission) [72]; ion transport proteins [73]; shank proteins (proteins that interact with the different glutaminergic receptors) [74]. It has also been shown that many genetic diseases predispose to the onset of ASD, including phenylketonuria [75], tuberous sclerosis [76], and neurofibromatosis [77]. Moreover, fragile X syndrome, which results from mutations in the fragile X mental retardation 1 (FMR1) gene, has been considered the most prevalent cause of monogenic ASD [78]. 

Several studies also correlate the deficiency of vitamin D [79], serotonin [80] and melatonin [81] during the gestation and early childhood stages with the onset of ASD. In addition, gene mutations predisposing to the development of ASD have also been correlated with parent seniority, with particular regard to the father. This observation was sustained by the increased risk highlighted in subjects bearing sperm genetic mutations [82]. 

Beyond these correlations, environmental factors also seem to be implicated in ASD onset. In fact, viral infections, maternal thalidomide use, alcohol consumption during pregnancy, gastrointestinal disturbances, the ingestion of mercury, retinoic and valproic acid and autoimmune and immune-related diseases, have also been considered as possible conditions to be involved in ASD development [83,84,85,86,87,88,89,90]. 

Moreover, an increased risk of ASD has been demonstrated amongst patients with CD, compared to healthy controls [10]. In addition, a correlation between ASD and an immune response to gluten has been suggested, also in the absence of CD but in the presence of AGA IgA and IgG [91], with 20% of ASD patients AGA IgG positive, [92]. However, AGA are not indicative of any autoimmune reaction. They appear only if the patients have been eating gluten, but they are not linked to any detectable adverse reaction to gluten. Thus, patients with NCGS, patients with irritable bowel disease (IBS) and CD, as well as individuals who are totally healthy, may all have AGA. AGA could not be relied upon to confirm or refute CD or NCGS diagnosis. However, the relationship between the ingestion of gluten, the presence of an immune or autoimmune response to it in AGA positive patients and the pathogenesis of ASD remains to be clarified. Among the gastrointestinal symptoms reported by parents of children with ASD and CD, there are diarrhea and inappetence. Besides, ASD children with CD could be asymptomatic at the time of the serological screening (AGA IgA/IgG, EMA and tTG) for the existence of true asymptomatic forms of CD, but this could also be because children with ASD are often unable to express gastrointestinal symptoms and systemic symptoms suggestive of CD, such as recurrent abdominal pain, abdominal distension and chronic fatigue. These clinical data suggest the usefulness of a serological screening for CD in young children with ASD, even in the absence of clear GI or systemic symptoms or other risk factors related to CD [93].

## 4. Opioid Hypothesis

Although the molecular mechanisms and genetic predispositions of CD and ASD have been well characterized, the events connecting these two disorders remain largely unknown. 

Evidence strongly indicates that an autistic behavior could be induced by an excessive and inappropriate brain opioid activity during the neonatal period [19,94]. Autistic patients show typical behavioral signs, such as the absence of social motivation, remoteness and solitary confinement. These signs have also been found in animals treated with exogenous opioids, suggesting their involvement in disease pathogenesis, known as the “Opioid Hypothesis”. This hypothesis was also confirmed by direct biochemical evidence, indicating abnormal levels of opioids in patients affected by autism and by the benefits provided in these patients by the therapeutic administration of naltrexone, a long-lasting agent able to block opioid receptors [95].

The first clinical observations of the co-association of CD and neurological disorders started with Bender in 1953 [96]. A possible explanation for the wheat and mental disorders connection is suggested by Dohan [97], who called neuroactive food antigen peptides “exorphins”, since they resembled the brain-reactive chemicals, endorphins. These molecules originate from the inadequate digestion of food proteins, which generates peptides called gliadomorphin (from gluten) and casomorphin (from casein). Gliadin digestion from wheat occurs through hydrolysis by gastric and intestinal enzymes, such as pepsin, leucine aminopeptidase and elastase, causing the release of immune-reactive and opioid-like peptide gliadinomorphin-7 (Tyr-Pro-Gln-Pro-Gln-Pro-Phe) [98]. A further digestion of these proline-rich molecules is carried out from the enzyme dipeptidyl peptidase IV (DPP IV) by cleaving N-terminal dipeptides with proline at the second position [99], releasing a tripeptide that acts as a selective competitive inhibitor for DPP IV [100]. These data suggest a key role for DPP IV in gliadin digestion and its deficiency/inactivity in genetically predisposed individuals could be involved in incomplete gluten breakdown, increasing the presence of exorphins [101,102,103,104]. Notably, DPP IV activity has been shown to decrease significantly in enterocytes of children with CD and this event has been correlated with mucosal damage [105]. Other proteins that are contained in dairy products and vegetables, such as soy and spinach, can be also converted into bioactive exorphins [106].

When produced, exorphins are absorbed across the gut barrier and, similar to endorphins, have the ability to bind to opioid receptors localized throughout the body. As they are also able to cross the blood–brain barrier, they interfere with neurotransmission, thus generating deleterious effects on brain maturation, attention, learning and public relations [96,107]. To support this evidence, some authors reported increased levels of exorphins in the bloodstream, cerebrospinal fluid and the urine of individuals affected by schizophrenia or autistic children [108,109,110,111,112]. In other studies, peptide levels correlated with symptom severity scores [113,114]. Furthermore, Dohan and Reichelt related the digestion of the principal component of bovine milk, casein, with the production of some exorphins involved in ASD pathogenesis, as well as in other mental disorders, such as schizophrenia and postpartum psychosis [97,112,115]. 

These exogenous opioid-like substances mimic endogenous opioid activity, influencing the intestinal transit and affecting behavioral traits, such as spontaneous behavior, memory, and pain perception in rodents. 

The highest behavioral influence was measured for casein and spinach-derived exorphins (B-casomorphin and rubiscolin, respectively) [116]. The effects of gliadin-derived exorphins were described by Takahashi et al. [117]. These authors reported that the intracerebral ventricular administration of gliadin-derived exorphin A5 induces anti nociceptive effects. In addition, when orally delivered, gliadin exorphin A5 modifies the learning and anxiety behavior in stressed mice. 

This indicates that orally delivered exorphins can influence both the peripheral and central nervous system, suggesting that gluten-derived exorphins have a strong opioid-like activity.

Beyond the production of exorphin by incomplete gluten digestion, an increased gut permeability may concur also with ASD development. It has been reported that both individuals with CD [54] and ASD [118] have increased gut permeability compared to the controls. Moreover, celiac patients have increased proportions of more harmful bacteria (especially *bacteroides* and *proteobacteria*), and decreased proportions of beneficial bacteria (especially *lactobacilli* and *bifidobacteria*). In addition, ASD children generally present decreased bacterial diversity compared to control populations with a significantly decreased *Bacteroides* to *Firmicutes* ratio [119]. Notably, different metabolite levels of bacterial origin, such as short chain fatty acids (SCFAs), indoles and lipopolysaccharides (LPS), were observed in the blood and urine of autistic children [118]. Thus, increased gut permeability in association with dysbiosis in individuals with CD may favor the passage of toxic compounds, as well as bacterial metabolite, through the intestinal barrier, which promote inflammation affecting the brain. 

**Figure 1:**
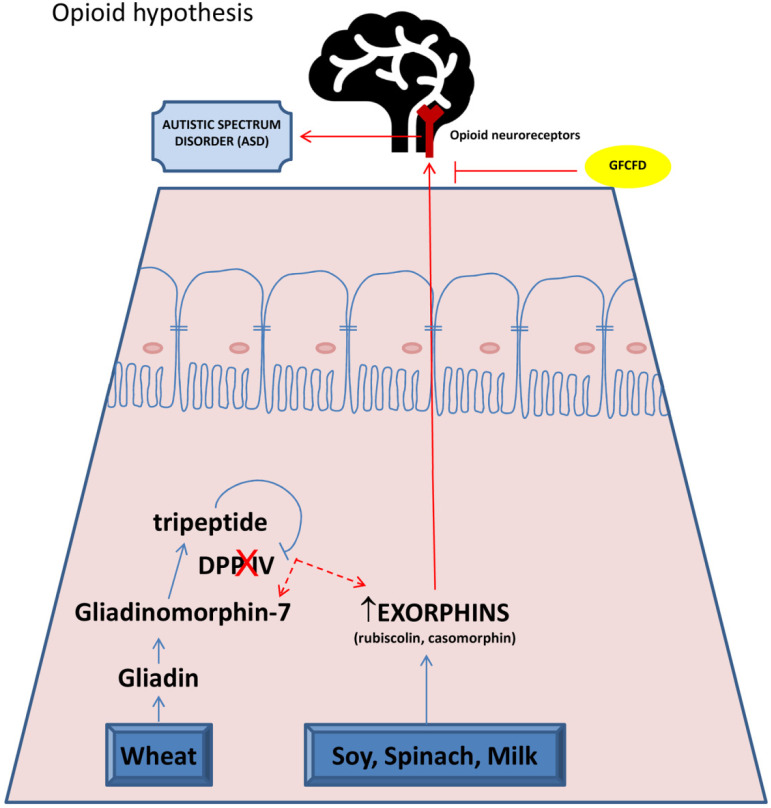
“Opioid hypothesis”. In celiac patients, the incomplete digestion of proteins derived by wheat and milk produces peptides called gliadinomorphin-7 and exorphins (gliadorphin, rubiscolin, casomorphin). The lack of protease DPP-IV favors the conversion of gliadinomorphin-7 in exorphins. Exorphins cross gut barrier and blood–brain barrier and are able to bind and stimulate opioid receptors in brain. These events could be responsible for disorders of attention, learning and public relations, typical of ASD. GFCFD: gluten free casein free diet.

Figure 1 describes the basis of opioid hypothesis correlating CD with ASD.

## 5. Oxidative Stress Hypothesis

Another interesting topic is represented by the many different studies that pinpointed a tight correlation between oxidative stress, ASD and CD. The accumulation of reactive oxygen species (ROS), as well as the impairment of protective antioxidant systems, can lead to oxidative injury in many different circumstances, affecting intestinal and extra intestinal areas [120], so that some authors speculated about the use of oxidative stress biomarkers for the management of CD. On the other hand, recently Waligora et al. have taken into account that neurological modifications occurring in ASD, such as intensity of emotional and behavioral symptoms, might be ascribed to oxidative damage [121] and considered ROS and biomarkers of oxidative stress as potential metabolic indicators for the development of appropriate programs of pharmacological therapy.

Oxidative stress is defined as a series of events resulting in an imbalance between the production of oxidative species and the activity of antioxidants [122]. Reactive oxygen species (ROS) and reactive nitrogen species (RNS) generation represent the main causal events of oxidative stress in the cells. ROS are reactive molecules and free radicals derived from molecular oxygen [122]. The main species include highly unstable oxygen free radicals, such as superoxide anion (O2•–), and hydroxyl radical (•OH), and more stable, freely diffusible non-radicals, including hydrogen peroxide (H_2_O_2_). Superoxide anion can react with nitric oxide (NO), which is produced by nitric oxide synthase (NOS), to generate the peroxynitrite anion (ONOO-), a reactive nitrogen entity with oxidative and nitrosative potential [122]. 

ROS are physiologically produced within the cells. The major cellular sources of ROS are mitochondria, where electrons that have escaped from the respiratory chain can react with O2 molecules to generate O2•– [123]. ROS can also be produced by the activity of different enzymes, such as NADPH oxidase, xanthine oxidase, lipoxygenases, and cyclooxygenases [124] or from the actions of different external factors, including pollutants, tobacco smokes, food and carcinogens [125,126,127,128].

ROS generated in the cells exert physiological roles or cause toxic effects in relationship with their levels [129]. At low doses ROS can function in the cells as signaling molecules by regulating different processes, including cell proliferation, gene activation and angiogenesis [130]. However, under oxidative stress conditions, overproduction of ROS can cause irreversible damage of macromolecules, including DNA and RNA, lipid peroxidation and amino acid oxidation with detrimental consequence for the cells [131]. To reduce the toxic effects resulting from the action of ROS, enzymatic antioxidant systems are active in the cells, involving oxido-reductase, such as superoxide dismutase, catalase, glutathione peroxidase (GPx) and glutathione reductase (GR) [132]. Furthermore, cells contain non-enzymatic antioxidant molecules, such as reduced glutathione (GSH), ascorbic acid, α-tocopherol and β-carotene. The antioxidant systems generally act by removing free radicals from cells, thus preventing their interaction with the biological macromolecules [132]. Therefore, their levels represent an important defense weapon against oxidative injury, especially for those organs, such as brain, where an elevated ROS generation was revealed. In fact, the brain has a high energy demand and consumes almost ten times more oxygen compared to other tissues [133]. Sustained mitochondrial activity can thus lead to increased ROS production because of electron escape from mitochondrial electron transport. ROS can, in turn, favor mitochondrial damage in neurons and the oxidative modification of macromolecules, leading to neuronal dysfunction [133,134]. The brain is highly susceptible to ROS damage because it contains high levels of polyunsaturated fatty acids, which undergo oxidation [134,135,136,137,138,139]. Lipid peroxidation leads to the production of toxic compounds, such as aldehydes, which in turn cause neuronal apoptosis. During the development and maturation of brain, antioxidants are necessary for neuron survival [140]. However, children (from conception through their infancy) have naturally lower levels of GSH than adults and, therefore, they are more susceptible to brain oxidative stress [141,142]. These data were confirmed by increased levels of environmental factors inducing oxidative stress in the placenta of their mothers.

An increase in oxidative and nitrosative stress and a decrease in the antioxidant capacity of the brain is involved in the pathogenesis of different neurological and neuropsychiatric disorders, including Alzheimer’s disease [135], Parkinson’s disease [135], ASD [134], schizophrenia [136], panic [137], obsessive–compulsive disorder [138], anxiety and depression [139]. These diseases are characterized by an increased inflammatory response caused by the accumulation of oxidized proteins or lipids in the brain. Concerning ASD, it has been reported that the autistic brain produces elevated levels of pro-oxidants, including xanthine oxidase and NO, and it also possesses reduced levels of protective antioxidants, such as GSH, SOD, GPx and catalase [134]. Moreover, some data also indicate a tight interrelationship between oxidative stress, mitochondrial dysfunction and ASD. This increase was also confirmed by meta-analysis data, sustaining that ASD could meet the criteria for a classical mitochondrial disease [143]. As it is well known, mitochondria represent the cell powerhouse [144,145] involved in breaking down nutrients to generate energy molecules to sustain all cellular processes. Pilot studies, performed using magnetic resonance spectroscopy, reported an impairment of mitochondrial energy metabolism in the dorsal prefrontal cortex [146] of young autistic patients, characterized by a dramatic decrease in the content of phosphocreatine and esterified ends (αATP + αADP + dinucleotides + diphosphosugars). On this subject, some findings accrued to the correlation between mitochondrial dysfunction and ASD, highlighting the accumulation of extended oxidative injury biomarkers, as well as some mutations in mitochondrial DNA that can be ascribed to gene deletions or uncontrolled replication [147].

Furthermore, some studies provided evidence that a consistent abnormal acyl-carnitine profile [148] occurs in ASD patients and over 80% of ASD children displayed a reduced function of the mitochondrial electron transport chain in lymphocytes. Moreover, mitochondrial dysfunctions, as well as defects in pyruvate dehydrogenase activity have been found in a recent work in autistic subjects [149]. However, since these data were obtained in a group of 12 children with ASD, the study deserves to be extended to a larger amount of cases to consider their possible involvement as new bio indicators of ASD.

Beyond ROS increase and mitochondrial dysfunction, the elevated production of NO also seems to exert a pathophysiological role in ASD [150]. NO, synthetized in the brain by neuronal Nitric Oxide Synthase (nNOS) from arginine, acts as a neuromodulator of the activity of different neurotransmitters, including glutamic acid and neuroammine [151]. However, it can also be produced in neuronal cells by inducible NOS (iNOS) under stress conditions, causing toxic effects in the cells. In fact, NO can interact with O2•–, yielding peroxinitrite (ONOO-), a reactive nitrogen species which favors the nitrosylation of macromolecules, leading to neuronal toxicity. A meta-analysis reported the presence of elevated levels of NO in the blood and urine of patients with neurodevelopmental disorders, especially hyperactivity disorder (ADHD) and ASD [152,153,154,155], probably caused by oxidative stress, which commonly represents a typical trait of these diseases [155,156]. An increase in NO production in ADHD and ASD subjects has been observed under chronic exposure to the widespread air pollutant nitrous oxide (N_2_O), which could have a pivotal role as a pathogenic factor in the development of ADHD and ASD in subjects with a reduced antioxidant capacity [157]. In fact, a prolonged N2O exposure, causing neural cholinergic inhibition, may adversely impact central NO metabolism and a fast-peripheral NO induction is required to restore central NO status [158].

On this subject, data reported in the literature also indicate the existence of an interaction between neuronal NOS (nNOS) and NADPH oxidase in mice, which was capable of affecting a battery of behavioral aspects, such as cognitive and social behavior [159]. The importance of this interaction was sustained by the observation that both nNOS and NADPH oxidase deletion favored the impairment of learning activity and social behavior in mice.

**Figure 2:**
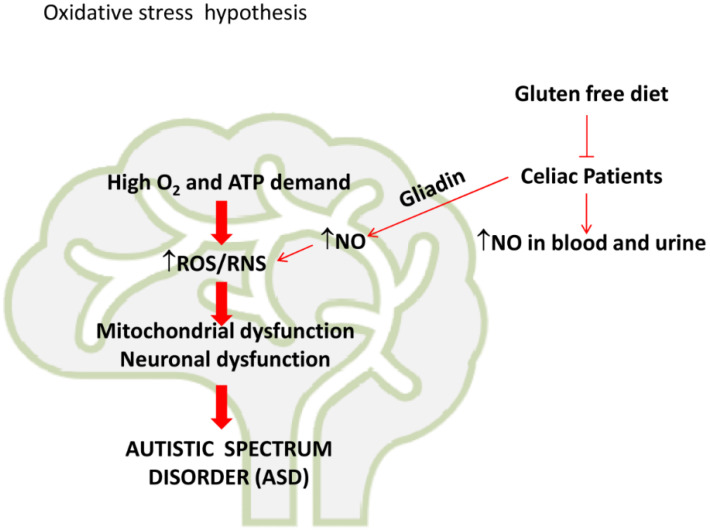
“Oxidative hypothesis”. The elevated oxygen consumption in brain favors ROS/RNS production. This induces mitochondrial and neuronal dysfunctions which could contribute to the development of Autistic Spectrum Disease. In celiac patients, gluten-derived peptides, such as gliadin, increase NO production and oxidative/nitrosative stress in brain. These events could contribute to the development of Autistic Spectrum Disorders in celiac patients.

Notably, increased oxidative and nitrosative stress could also be a possible explanation for the connection between ASD and CD, as shown in Figure 2. 

Interestingly, it has been reported that oxidative stress is implicated in the pathogenesis of CD [160]. Several in vitro studies demonstrated that the exposure of cells to gliadin increases the level of free radicals [161]. In addition, gluten exposure can induce an intracellular oxidative imbalance in CD patients. In line with these findings, Stojilikovic et al. [162] demonstrated that the levels of lipid peroxidation products increased in children with CD, whereas the levels of GSH and the antioxidant enzymes GPx and GR are significantly reduced. Furthermore, as already demonstrated by clinical and in vitro findings [163], gluten consumption in children with ASD induces an increase in N_2_O-induced oxidative burden and a consequent increase in the levels of NO in the blood and urine, contributing to the symptoms of gluten intolerance. This fact is consistent with the role of NO as a regulator of intestinal inflammation and a modulator of gut microbiome health [163,164]. NO production in CD has been correlated with gliadin production. Notably, Maiuri [165] shows that gliadin increased iNOS protein expression in RAW 264.7 macrophages, stimulated with IFN-α, and that this increase is correlated with the activation of NF-kB. Thus, gliadin production in CD patients can favor oxidative stress-induced brain damage in ASD patients. To support the role of oxidative stress in the correlation between CD and ADHD/ASD, Fluegge [166] shows that a gluten free diet could be recommended for certain ADHD/ASD patients with increased levels of blood and urinary NO. However, further clinical studies should be performed to strengthen these correlations. 

A gluten-free diet (GFD) is the only treatment for CD and this has been reported to reduce the risk of psychiatric disorders [167]. ASD patients are often advised to adopt a GFD to reduced behavioral problems. However, scientific evidence suggests that the use of gluten-free diets in the treatment of autism is weak and there is no rationale for doing so unless the patients have been tested for CD prior to adopting a GFD. Many small studies examined the effects of the elimination of gluten from the diet on autistic symptoms, although the majority of them evaluated the gluten-free casein-free diet (GFCFD). In fact, the most popular dietary intervention is a gluten-free and casein-free diet, which is based on the theory that autistic patients have a “leaky gut”, allowing peptides to leak out of the intestines into the bloodstream. Therefore, a diet excluding casein and gluten can be useful to reduce the levels of opioid peptides in autistic patients, thereby helping to improve autistic symptoms [126,139,140]. The use of a gluten-free and casein-free diet is often reported by parents of autistic patients, as described in two Cochrane Reviews published in 2004 and 2008 [141,142]. The autistic patients were children, adolescents and adults, and the types of treatment evaluated were gluten-free, casein-free, or gluten- and casein-free diets vs. placebo/no treatment and gluten-free diet vs. casein-free diet. The efficacy of the diet was measured in terms of urine peptide levels, behavioral evaluation, linguistic and motor ability, cognitive functioning and general quality of life. A trial with seven children affected by ASD on six months of GFD, without the control group, did not demonstrate any relationship between gluten and ASD symptoms [168]. Another small study without a control group reported an improvement in autistic-typical behavior, without bringing any positive change in the levels of urinary peptides [169]. Recently, 80 ASD children placed in a randomized and controlled trial demonstrated an improvement in intestinal symptoms and communication and language [170]. Furthermore, a single-blind trial with 20 children affected by ASD on a GFCFD showed significant improvements in autistic-typical behavior and communication [171], while another double-blind cross over trial with 15 ASD children demonstrated no statistical significant differences among the treated and not treated group [107,172]. Finally, three trials performed on casein and gluten in children during GFCFD, randomly receiving gluten or casein placebo foods, demonstrated no difference in behavior and intestinal symptoms between the two groups [173,174,175]. However, considering the use of GFD for conditions other than celiac disease, scientific evidence suggests that it is necessary to evaluate the real benefits. The use of GFD or GFCFD in the treatment of autism is weak, still controversial, and there is a need to explore this field more deeply. GFDs are frequently poor in fiber, micronutrients, such as folate, Vitamin D and B12, and mostly in minerals, including zinc, calcium, magnesium, iron, while being too rich in saturated fat and sugar [176,177,178,179]. The Nurses’ Health Study and Health Professional Follow-Up Study highlighted an inverse correlation ratio between gluten intake and type 2 diabetes arising in people not affected by CD [180,181], even if it may result in a reduction in whole grains with a great cardiovascular benefit [180]. Additionally, people on GFDs were often found to have higher levels of arsenic in the urine and mercury in the serum, probably due to a wider consumption of fish and rice containing high amounts of these metals and, moreover, they often complained about anxiety [182,183]. Finally, a gluten-free and casein-free diet could have many disadvantages, since this diet costs more than the standard one and, finally, it is often difficult to change autistic children’s dietary habits. Nutrients could have broader effects than drugs, so it is appropriate that parents apply these dietary interventions with the help of a nutritionist or other qualified practitioners, as if they were a real therapeutic treatments [144,145]. Thus, the elimination of gluten from the diet of ASD patients with or without gastrointestinal symptoms, but not with a biopsy-diagnosed CD, is a thing to do very carefully, computing the real advantages. 

## 6. Conclusions

Celiac disease is an immune-mediated pathology triggered by gluten-derived proteins, which is characterized by a chronic inflammation of the intestinal epithelium. Beyond gastrointestinal symptoms, celiac patients can be affected by neurological and psychiatric disorders, including epilepsy, schizophrenia and ASD. Although the correlation between CD and ASD is supported by different clinical studies, the exact mechanism, on the basis of this correlation, remains unknown. Data presented in the literature correlating CD with ASD highlight how gluten-derived peptides can bind and stimulate endogenous opioid receptors. This effect could explain the defects in the brain maturation, attention, learning and public relations of autistic patients. Moreover, several clinical and in vitro studies supported the hypothesis that oxidative and nitrosative stress associated with gluten-related disorders can favor mitochondrial damage and neurological dysfunctions, which are involved in the pathogenesis of ASD. Therefore, we wondered whether a possible correlation exists between these two hypotheses. Notably, the study of Huang [184], supporting a role for NO in the modulation of μ-opioid receptors, the class of opioid receptors involved in ASD [94], suggests a possible relationship between the two hypotheses. However, other studies need to be conducted to ascertain this link and establish whether oxidative stress and brain opioid activity can be at the root of these pathologies connecting each other. 

## Figures and Tables

**Table 1:** Celiac Disease: risk factors and related conditions.

What Is Celiac Disease (CD)	Risk Factors for CD	Conditions Related to CD
Celiac disease (CD): Immune response to gluten protein [1,2]; CD is present in 1% of the general population [3,4,5].	Chronic inflammation [5,6,7] Genetic predisposition, infant feeding and birth with caesarean section [38] Autoimmune disease [7] Down, William and Turner syndrome [7] Cystic fibrosis [7] Gut–mucosal barrier dysfunction [54].	Gastrointestinal conditions: weight loss, fatigue, diarrhea and bloating [49] Neurological conditions: epilepsy, ataxia and migraine [11] Autistic Spectrum Disorder [66] Neuropsychiatric conditions: epilepsy, ataxia and migraine [11] Schizophrenia [67]

## Abbreviations

ADS	Autistic Spectrum Disorder
CD	Celiac Disease
CLDNs	Claudins
CXCR3 receptor	Chemokine receptor 3
DPP IV	dipeptidyl peptidase IV
GFCFD	gluten free casein free diet.
GSH	glutathione
GPx	glutathione peroxidase
GRD	Gluten Related Disorder
iNOS	inducible Nitric Oxide Synthase
•OH	hydroxyl radical
H_2_O_2_	hydrogen peroxide
ADHD	hyperactivity disorder
HLA	Human Leukocytes Antigens
IFN-α	Interferon alpha
IFN-γ	Interferon gamma
IL17A	Interleukin-17A
IL-8	Interleukin-8
IBS	Irritable bowel syndrome
nNOS	neuronal Nitric Oxide Synthase
NO	nitric oxide
NOS	Nitric Oxide Synthase
N_2_O	nitrous oxide
NCGS	Non-Celiac Gluten Sensitivity
ONOO^-^	peroxynitrite anion
RNS	reactive nitrogen species
ROS	reactive oxygen species
O2•	superoxide anion
TJ	tight junctions
Th	T helper
TNF-α	tumor necrosis factor alpha
WA	Wheat allergy
